# Deep brain stimulation, lesioning, focused ultrasound: update on utility

**DOI:** 10.3934/Neuroscience.2023007

**Published:** 2023-04-26

**Authors:** Akshay Reddy, Mohammad Reza Hosseini, Aashay Patel, Ramy Sharaf, Vishruth Reddy, Arman Tabarestani, Brandon Lucke-Wold

**Affiliations:** 1 College of Medicine, University of Florida, USA; 2 Feinberg School of Medicine, Northwestern University, USA

**Keywords:** deep brain stimulation, lesioning, focused ultrasound

## Abstract

Procedures for neurological disorders such as Parkinsons Disease (PD), Essential Tremor (ET), Obsessive Compulsive Disorder (OCD), Tourette's Syndrome (TS), and Major Depressive Disorder (MDD) tend to overlap. Common therapeutic procedures include deep brain stimulation (DBS), lesioning, and focused ultrasound (FUS). There has been significant change and innovation regarding targeting mechanisms and new advancements in this field allowing for better clinical outcomes in patients with severe cases of these conditions. In this review, we discuss advancements and recent discoveries regarding these three procedures and how they have led to changes in utilization in certain conditions. We further discuss the advantages and drawbacks of these treatments in certain conditions and the emerging advancements in brain-computer interface (BCI) and its utility as a therapeutic for neurological disorders.

## 1. Overview

### 1.1. Deep Brain Stimulation

Deep Brain Stimulation (DBS) is a technique that delivers continuous electrical stimulation through implanted electrodes, which are usually connected to an internalized device such as a neuropacemaker or stimulation [1]. Although the most common utilization of DBS is pain alleviation and treatment of movement disorders, the unique advantage of DBS is the ability to adapt to varying patient needs by altering the stimulator's frequency delivered during different intervals [1],[2]. By additionally manipulating the placement of the implanted electrodes, DBS offers a broad range of therapeutic applications in both the general clinical setting, as well as in the minimally invasive neurosurgical setting. Most commonly, DBS is used to target neurological structures including the subthalamic nuclei (STN), globus pallidus interna (GPi), and thalamus [3],[4]. Although each of these structures may be targeted via DBS to provide therapy for tremors, DBS can also be used to specifically target the STN to achieve therapeutic treatment for patients with Parkinson's disease or patients with either dyskinesia or dystonia [4]. While administration of L-DOPA remains the most effective therapy for patients with Parkinson's disease, greater therapeutic efficacy can be achieved by additionally utilizing DBS in conjunction [5]. For patients who are only mildly responsive to L-DOPA, the GPi may be targeted as a treatment approach for patients with Parkinson's disease who also demonstrate a considerable decline in cognitive function [4],[5]. Conversely, the thalamus may be targeted to provide therapeutic relief of essential tremors, as the thalamus is not involved in rigidity or postural control [4]. DBS has also demonstrated therapeutic efficacy across a broader range of clinical applications, including treatment for drug addiction, Tourette syndrome, mood disorders such as depression, and obsessive-compulsive disorders [1]–[4].

### 1.2. Lesioning

Lesioning is a surgical technique where small areas of damage are made to the brain, usually targeting cells that control movement with the therapeutic goal of treating movement disorders [6]. During the 1950s through the 1960s this was a common method of treatment for movement disorders, but has shown mixed results, and today is reserved for patients with severe movement disorders who do not respond well to medications, cannot tolerate the side effects of medication, or who are not good candidates for DBS [7],[8]. The types of lesioning procedures are differentiated primarily by the targets of the procedures. In a pallidotomy a lesion is made within the GPi which tends to be overactive in patients with Parkinson's, however in patients resistant to L-dopa, lesioning may not be an efficacious treatment [8]. A thalamotomy targets the thalamus due to its role in controlling motor responses and is useful for the treatment of dystonia or one-sided Parkinson's tremors [6],[7]. A subthalmotomy targets the subthalamus and is the rarest form of lesioning procedure. The lesion itself can be created in a variety of methods such as radiofrequency using high-frequency radio waves, radiosurgery using radiation, or even with probes filled with liquid nitrogen that are then inserted into the brain [7],[8].

### 1.3. Focused Ultrasound

Focused ultrasound (FUS) is a minimally invasive technique that leverages Ultrasound technology to deliver targeted sound waves to produce therapeutic chemical and/or mechanical changes [9]. In contrast to ultrasounds, where sound waves are utilized to produce diagnostic images FUS instead relies on higher sound wave frequencies and intensities which are converged to produce a beam of energy that can be specifically targeted to achieve chemical, thermal, or mechanical work in deep tissue [9],[10]. The development of the FUS technology thus provides a minimally invasive alternative to more invasive surgical approaches, whilst also allowing for more localized and precise delivery of therapeutic treatment to optimize the balance between therapeutic efficacy and damage to surrounding tissue [9]–[11]. As such, FUS is currently being leveraged in a growing number of therapeutic applications including thermal ablation of malignant and/or nonmalignant growths, blood-brain barrier disruption, and neuromodulation [9]–[15]. Additionally, given the level of precision that can be achieved with FUS, the technology can be utilized to induce the formation of pores on cell membranes to enhance drug delivery [9],[10]. More specifically, FUS has previously been utilized successfully in the therapeutic management of conditions such as essential tremors, tumors, embolisms and thromboses, and synucleinopathies – including Parkinson's Disease [9],[10],[15],[16].

### 1.4. Utilization

These respective procedures, as aforementioned, are widely applicable in a series of neurodegenerative and neurological diseases, but to a varied extent. Different conditions have different primary methods of treatment and use of these specific procedures. Although utilization of these methods is often interchangeable, various studies have shown that different pathologies are often better managed using one treatment over another.

### 1.5. Parkinson's Disease

Parkinson's Disease (PD) is a neurodegenerative disorder that is classically described by a progressive deterioration of central motor control, including symptoms of bradykinesia, rigidity, and resting tremors [17]. Additionally, PD is often associated with nonmotor symptoms, such as Alzheimer's, psychiatric changes, and disturbances in sleep [18]. It is well-understood that PD primarily arises from the death of dopaminergic neurons in the substantia nigra of the midbrain, yielding an insufficient dopamine supply to the globus pallidus of the basal ganglia [19]. Additionally, PD neurodegeneration is not only limited to dopaminergic structures, as there is evidence of Lewy-Body formation and neuronal death affecting the cholinergic system central to the nucleus basalis of Meynert, the entorhinal cortex, and autonomic nervous system [20]. The loss of dopaminergic input contributes to motor symptoms experienced, while cholinergic loss is thought to be associated with nonmotor comorbidities, such as dementia [19],[20]. Several therapies have been leveraged to restore the dopaminergic supply in PD patients, including the frontline treatment of levodopa and other dopamine agonist medications [21]. PD has been treated recently by DBS, lesioning, and FUS.

Recently, Deep Brain Stimulation (DBS) has emerged as a potentially viable intervention for advanced PD resistant to levodopa therapy [21]. In the case of PD, the STN has been seen to be an effective target for improving patient symptoms along with GPi [22],[23]. Recent studies have not shown long-term significant differences in efficacy in targeting the STN or GPi through DBS. However, analyses have indicated that off-drug motor symptoms have more improvement post-STN-DBS [24]. Both targets have been seen to be effective in the long term, but GPi has been seen to have a larger impact on dyskinesia and STN-DBS is associated with a larger decrease in dopaminergic medications [25]–[28]. Uniquely, studies have shown that GPi-DBS is potentially a better target for patients with gait difficulty as well [24]. The utilization of DBS to target both STN and GPi evolved from an initial targeting of the ventralis intermedius nucleus (VIM). Although VIM targeting provided sufficient tremor control, it failed in relieving other symptoms over time, leading to exploring other targets [29]–[31]. Uniquely, recent research has shown that in the off-drug condition group patients' motor symptoms were significantly improved, while in the on-drug condition, dyskinesias were improved [22],[32]. Furthermore, when switching DBS off in the STN, the UPDRS motor score worsened within two hours, while turning it back on improved all scores at a faster rate than they worsened [32],[33]. Lastly, DBS utilization has been seen to improve the quality of life (PDQL) more so than motor symptoms, by improving all aspects of PDQL by 43% [32],[33].

Lesioning surgeries (LS), such as pallidotomy, thalamotomy, and subthalamotomy are also available in the treatment of PD, albeit to a lesser extent. Surgical procedures tend to be included as an option when patient symptoms are not able to be managed effectively by oral medications alone. However, utilization of LS, although less than DBS, is still prevalent in less developed countries due to a lack of training, finances, and limited awareness [34]. The smaller expenditure and decreased need for post-operative care along with improvements in imaging make LS a viable alternative option to DBS. Further innovation since the 1990s also introduced radiofrequency (RF), magnetic resonance-guided focused ultrasound (MRgFUS), laser interstitial thermal therapy (LITT), and MRI-guided high-intensity focused ultrasound (HIFU) [35]. MRgFUS has become popular too due to its lack of invasiveness since there is no incision, less ionizing radiation, and faster results [35],[36]. However, limitations include the dependence on factors such as the patient's skull characteristics (e.g. thickness) and longer operating times. Currently, MRgFUS thalamotomies are approved but further investigation is undergoing for its use in pallidotomies and subthalamotomies [36],[37]. Thalamotomy is primarily considered for tremors and pallidotomy has been seen to further improve bradykinesia and rigidity [35],[38]–[41]. Recent studies have shown LS of the STN is associated with improvement in motor symptoms without prolonged adverse effects making it a potential surgical option as well [38]–[40].

### 1.6. Essential Tremor

Like PD, Essential Tremor (ET) is also a neurological condition characterized by a progressive decline in motor control [42]. ET is differentiated from Parkinsonian tremors in that ET typically presents actively and bilaterally in the hands and may additionally involve the head and voice at more advanced stages, whereas PD tremors are commonly asymmetrical, present at rest, and do not involve the head [16],[42]. Furthermore, ET is associated with mild cognitive impairment, commonly affecting the attention and working memory of patients [16]. The contrast between the nature of ET and PD associated tremors potentially arises from the different pathophysiology of the two conditions. Primarily, it is thought that ET arises due to dysfunction in the gamma-aminobutyric acid (GABA) system secondary to inhibitory Purkinje cell death in the cerebellum, which results in upregulated oscillatory activity in the thalamus and thalamocortical circuit [43]. However, there is also consideration of the basal ganglia having a role in the onset of tremors related to ET through network interactions with the cerebellothalamocortical circuit [44]. Typically, medical treatment for ET symptoms includes primidone, an anticonvulsant, and propranolol, a beta-adrenergic blocker [16],[45]. For drug-resistant ET or cases where patient quality of life is severely impacted, interventional therapies—thalamic lesioning and more recently, DBS and MRI-guided high-intensity focused ultrasound specifically targeting the thalamus—are indicated to lessen symptoms [16].

DBS use in ET is associated with effective tremor reduction and many studies are currently focused on optimal utilization and targeting [46],[47]. The classical target for DBS in ET has been the VIM as unilateral VIM-DBS has been seen to suppress tumors and has the potential for suppressing axial tremors [48]. VIM targeting is also associated with improved contralateral distal limb tremor and voice tremor [48]. Recent research has also shown the potential of the posterior subthalamic area (PSA) and the caudal zona incerta (Zi) as therapeutic targets associated with tumor reduction [49],[50]. Both the PSA and Zi are novel locational approaches to ET DBS which can be more effective than VIM targeting, as seen through further reduction of tremor in several instances in comparison to VIM [49],[51]. Furthermore, bilateral procedures have been more effective for midline tremors while unilateral procedures have been more effective for contralateral hand tremors [52]. Adverse effects of ET DBS tend to be more frequent during bilateral use than unilateral use and the most common adverse event tends to be dysarthria [52].

Lesioning procedures in ET include radiofrequency (RF), thalamotomy, and magnetic resonance-guided focused ultrasound (MRgFUS). MRgFUS typically targets the VIM of the thalamus after the patient is usually screened using a skull density ratio (SDR) to be approved for the procedure [53]. Studies have shown the most effective utilization of MRgFUS is a lesion in the posterior section of the VIM close to the boundary with the ventralis caudalis (VC) nucleus [54]. However, slight error leading to lesioning closer to the VC has a significantly higher chance of contralateral sensory disturbance [54],[55]. A recent randomized control trial (RCT) using sham MRgFUS control shows a significant reduction in contralateral arm tremor and improved quality of life in patients with VIM lesions, displaying its efficacy and justified utilization in ET [11]. Within the RCT, the common adverse events were gait ataxia and paresthesia, of which less than 12% lasted at 12-month follow-up. As further studies have shown significant improvement over a longer period, the utilization of focused ultrasound, along with other lesioning treatments, has become more popular [56].

### 1.7. Obsessive Compulsive Disorder

Obsessive Compulsive Disorder (OCD) is a neuropsychiatric disorder that results in patients experiencing frequently repeated intrusive thoughts and urges, otherwise known as obsessions, as well as recurrent, often involuntary behaviors described as compulsions [57],[58]. These chronic symptoms have a significant impact on patient quality of life, yet their clinical diversity and variable manifestations make early diagnoses challenging, thus restricting access to early treatment [58]. Another important factor adding to the complexity of OCD is the limited knowledge regarding its pathogenesis. Currently, neuroimaging studies utilizing fMRI have demonstrated evidence of altered functional connectivity between the rostral anterior cingulate cortex and dorsolateral prefrontal cortex in patients with OCD [59]. This is in line with the hypothesis that OCD potentially arises from imbalances in corticostriatal network interactions, affecting structures such as the orbitofrontal cortex, GPi, and striatum [59],[60]. Typically, first-line treatment for OCD includes medication, mainly serotonin reuptake inhibitors with antipsychotic augmentation [61], and cognitive behavior therapy in the form of exposure with response prevention [62],[63]. Keeping in trend as previously discussed with PD and ET, surgical intervention is indicated for only the most drug-resistant and debilitating cases of OCD. Modern surgical procedures to treat OCD include stereotactic lesioning and DBS [64].

DBS utilization, since approved for more severe and chronic cases of OCD by the FDA in 2009, has been seen to alleviate symptoms through modulating disturbances in underlying circuitry. Initially, DBS had a broader target in the entire dorsoventral length of the anterior limb of the internal capsule (ALIC) [65]. More recently the focus has been on the ventral capsule/ventral striatum (VC/VS) of the striatal region, which has shown a significant decrease in the Yale-Brown Obsessive Compulsive Scale (Y-BOCS) of OCD severity [66]. Recent findings suggesting more posterior targeting while at lower stimulation amplitudes is effective led to the eventual FDA approval of ALIC DBS [67]. Currently, the two focused targets are the striatal region, including the ALIC, VC/VS, the nucleus accumbens (NAc), and the ventral caudate nucleus. The second target area is the STN [68],[69]. More recently, targeting the anteromedial STN (amSTN) has seen the best response rates in RCTs and a larger effect on OCD symptoms on a long-term scale (46 months) [70]. In the various clinical trials investigating the efficacy of DBS in OCD, the reduction in OCD response tended to be roughly 45–48% [71]. Adverse events are frequent but tend to be relatively mild and a majority resolve within a month, making the utilization of DBS in OCD relatively successful [71].

LS in OCD tends to be used only in the most severe cases when other options have been considered. Currently, the four primary targets for LS are the anterior capsule (AC), cingulated gyrus (CG), subcaudate tractotomy, limbic leucotomy, and NAc. Subcaudate tractotomy and limbic leucotomy are less common but involve more extensive lesions, urging caution in utilization [64]. Recently, using MRgFUS for bilateral thermal lesioning of the ALIC has been seen to decrease Y-BOCS scores over two years [72]. Utilization of MRgFUS further improved symptoms within a week and uniquely had a lack of major adverse events [72]. Compared to traditional procedures, MRgFUS non-radiation ablation and no incision make it a promising innovation in treating OCD. The ability to use advances in imaging to verify lesion size and ablation temperature allows for precision targeting of the ALIC, minimizing complications [72].

### 1.8. Tourette Syndrome

Tourette Syndrome (TS) is a childhood-onset neurodevelopmental disorder that is commonly described by repetitive, rapid, and stereotyped tics of either a vocal or motor nature [73],[74]. These tics normally begin around the age of eight years old, reach a maximum in occurrence around adolescence, and, for about 50% of patients, subside into adulthood [75]. The exact pathophysiology of TS and its related tic symptoms remain unknown, but basal ganglia dysfunction is implicated to be a root cause due to its inhibitory outputs on motor nuclei of the thalamus and motor pattern generators of the cerebral cortex [74]. Typically, first-line medication for TS consists of alpha-adrenergic blockers, such as clonidine and guanfacine, and benzodiazepines, such as clonazepam and diazepam [74]. However, for more severe adult cases where medication cannot alleviate symptoms, DBS targeting the GPi and thalamus is now indicated at an increasing rate for treating TS [74],[75].

DBS of basal ganglia-thalamocortical networks has recently been seen as an effective intervention for TS. The primary target for TS DBS has been the thalamus due to its location between both the cerebral cortex and subcortical structures [76]. Utilization provides a sufficient improvement in TS motor and phonic tics [77],[78]. However, targeting of the thalamus is associated with adverse effects such as blurry vision and dysarthria [78]. The GPi is another target for TS DBS primarily in the setting of severe and refractory TS [79]. Utilization of GPi targeting through a bilateral approach is associated with a significantly reduced tic severity [80]. Recent research has also found the STN as an effective target, as it has been associated with a potentially faster relief in tics in comparison to other targets due to its modulation of both limbic and sensorimotor areas [76].

### 1.9. Major Depressive Disorder

Major Depressive Disorder (MDD) is among the most common psychiatric disorders and is characterized by altered or depressed mood, decreased pleasure from conducting daily activities, and cognitive impairment [81],[82]. It is thought that the causes of MDD are multifactorial, involving genetics and environmental factors, as well as alterations in functional neural circuits [81]. Of special consideration is the “affective-salience circuit,” which is involved in goal-directed behavior and includes brain structures such as the dorsal cingulate, amygdala, anterior insula, and ventral striatum [83]. Abnormally high activity in the dorsal cingulate, amygdala, and anterior insula has been demonstrated in MDD patients using neuroimaging studies [83],[84]. MDD is typically treated with selective serotonin reuptake inhibitors (SSRIs), serotonin and norepinephrine reuptake inhibitors (SNRIs), and less commonly, monoamine oxidation inhibitors (MAOIs) [85]. Recently, DBS has been tested as an alternative treatment for severe depressive symptoms, as well as bipolar disorder with depressive moods [73],[85].

The use of DBS for the treatment of MDD has expanded and studies on its efficacy can be stratified by the location of testing [86]. The subcallosal cingulate gyrus (SCG) has had the largest amount of study conducted on its efficacy and shows promising results on response and reduction of depressive symptoms. DBS treatment is generally well-tolerated and even within chronic applications of DBS in the SCG over two years showed no hypomanic or manic episodes in the participants [86],[87]. Other areas targeted by DBS for the treatment of MDD are the ventral striatum (VS), nucleus accumbens (NAc), as well as the medial forebrain bundle(tMFB). All these targets show minimal risk for adverse side effects and greater recovery and response rates compared to remission [86]–[89].

Focused ultrasound (FUS) as well as general radiofrequency and radiosurgery lesioning procedures are quickly becoming more popular, with FUS being a popular noninvasive therapeutic choice in patients with treatment-resistant resistant depression [90],[91]. Limited therapeutic options in the field of treatment for medication-resistant patients with MDD have made this a topic of growing study [91],[92]. Anterior cingulotomies, subcaudate tractotomies, limbic leucotomies, and anterior capsulotomies hold the bulk of studies on efficacy showing approximately a third to a half of patients who underwent ablative procedures achieving treatment response and/or remission [91]. Despite some similarities, the safety profiles of ablation and stimulation differ. In terms of the procedures, both methods require a cranial window and transcortical transgression, unless GKRS or MRgFUS is used. However, DBS can have complications, such as device malfunction, breakage, disconnection, and infection. Regardless, adverse events (AEs) reported for psychiatric diseases have been comparable for both procedures. Both procedures have shown temporary perioperative AEs, such as headaches, confusion, and incontinence. Although uncommon, serious AEs like postoperative seizures, cognitive impairment, and suicide have been reported for both ablative and stimulation procedures. Electrical stimulation in DBS can be adjusted, offering a potential advantage in reducing and possibly eliminating some of these AEs.

## 2. Advantages, disadvantages, and innovations

### 2.1. Deep Brain Stimulation

Deep brain stimulation (DBS) is a surgical technique used to modulate neurons through circuits in the brain. The modern DBS system consists of an intracranial electrode, an extension wire, and a pulse generator. DBS is now being investigated for a variety of other diseases, including neuropathic pain, cluster headache, epilepsy, obsessive-compulsive disorder (OCD), depression, Alzheimer's disease, Tourette syndrome, addiction, anorexia nervosa, and schizophrenia [93].

### 2.2. Advantages

DBS has several advantages over lesioning, including its reversibility, ability to program the stimulator, and capability to perform bilateral procedures without inducing pseudobulbar and other deficits. Deep brain stimulation is a reversible process. The device can be removed without causing any permanent neurological damage in the future if, following the collection of more data, a better treatment is found [94]. DBS is equivalent to surgical lesioning (pallidotomy) in cases of unilateral brain stimulation and superior when bilateral devices are placed, in terms of efficacy and side effects, compared to unilateral brain stimulation. Due to the high rate of side effects associated with bilateral surgical lesioning, the procedure is no longer performed [95].

### 2.3. Limitations

DBS costs are higher than lesioning, requiring more patient commitment and follow-up visits to specialized centers for optimization. Patients living in developing countries where DBS is unavailable or if the patient lives far from a center may face this problem. The cost is reduced in the bilateral subthalamic nucleus stimulation studies because the medication is reduced over five to seven years [96],[97]. Despite its benefits, DBS has some limitations, including the size and life of the batteries and the need to replace them frequently. However, multiple manufacturers now offer DBS technology on the global market, resulting in increased international competition and faster progress [98],[99]. Complications that may occur to a patient with a DBS system include neurostimulator battery depletion, erosive skin, infection of the skin or hair, adhesions of scar tissue, movement of the brain wire, hardware malfunction, battery failure, behavioral changes, and lack or loss of efficacy [100].

## 3. Complications

### 3.1. Neurostimulator battery depletion

The depletion of neurostimulator batteries should be prevented since patients who suddenly lose stimulation may suffer adverse effects. Typically, batteries last between 3 and 5 years, depending on the stimulation intensity. Recording therapeutic stimulation settings are important for all patients, especially while traveling. When symptoms change, hardware system and neurostimulator battery checks should be performed, as well as during the battery's expected lifespan, typically every six months. Replacements of neurostimulators are usually performed on outpatients under general anesthesia [101].

### 3.2. Skin erosion

Skin erosion is more likely to occur in areas where hardware protrudes, tensioning the skin overlying the incision or around thinned skin. An eroded skin surface may be erythematous, painful, scabby, or itchy. Untreated erosion can eventually result in hardware infection and the need to remove the entire implant based on its location [102]. Before skin integrity is disturbed by skin erosion, surgical skin revision is required to prevent hardware infection [103].

### 3.3. Infection of the skin or hair follicles

The DBS system may need to be removed if a skin infection or hair follicle infection occurs near the implanted device. Erythema, drainage, and fever are typical hardware infection symptoms; malaise, chills, and fever are late manifestations that indicate systemic involvement. Subcutaneous hardware infection can cause brain abscess, so rapid assessment and treatment are necessary. Parenchyma displacement is not known to occur during intracranial hardware extraction.

### 3.4. Implant infection

The infection rate for chronic DBS patients ranges from 5–10%, adding to the importance of preventing infection of implantable devices. Pacemaker infections can already be prevented using antibacterial envelopes. According to a recent randomized controlled study, antibacterial envelopes significantly reduce infection rates in people with cardiac implantable devices. Also, antibiotic coatings could prevent infection and the subsequent removal of neurostimulation systems [104]–[106].

### 3.5. Scar tissue adhesions

Scar tissue adhesions near extension wires may limit head movement or cause pain, which may require repositioning of the extension wire or scar tissue placement. During the postoperative period, cervical range-of-motion activities are crucial to avoiding this problem [107].

### 3.6. Brain Wire Migrates

In rare cases, the brain wire migrates, and symptoms can return abruptly. If the wire has moved significantly, a skull radiograph can confirm migration or additional imaging may be needed to assess for a change in tip location. The wire location is often documented with postoperative brain imaging, as even a few millimeter movements can result in suboptimal or lost control of symptoms [108]. Symptoms of hardware breakdown may include sudden electrical shocks, paresthesias, burning sensations, and muscle contractions. A change in body position that is associated with new-onset symptoms should be considered hardware malfunction until proven otherwise. Clinicians with deep brain stimulation expertise should assess suspected hardware problems. To determine whether wires are intact or damaged, the clinician can measure both resistance and current [109].

### 3.7. Behavioral changes

DBS may cause behavioral changes in some patients including anxiety, depression, hypersexuality, hypomania, apathy, personality changes, and suicide [110].

### 3.8. Lack of efficacy

All procedures in the protocol must be followed correctly for DBS to be effective. Failure to achieve efficacy may be caused by incorrect placement of lead wires in the brain, malfunction of the lead wires, inadequate medication, and misdiagnosis [111].

### 3.9. Acute efficacy loss

A variety of factors can cause acute efficacy loss. Wire breaks or depleted batteries can cause the device to malfunction. Recent medication changes, systemic illnesses, or recent stimulation parameters can acutely alter patients' neurologic symptoms.

### 3.10. Focused Ultrasound

Although DBS is an effective neuromodulation method, it is also associated with surgical complications. Thermal lesioning is possible with MRgFUS without incision, which is advantageous since it reduces surgical complications, although the effects are irreversible.

The FUS procedure does not require a cranial incision or anesthesia, making it a suitable alternative for patients who are not comfortable with skull holes. Furthermore, non-incisional therapies appear to have a lower risk of hemorrhage [112].

As for intraprocedural monitoring, FUS is monitored in real-time before final lesions are delivered. In this manner, it may be possible to target therapies in a more precise manner that may maximize their effectiveness as well as minimize their adverse effects [113].

This technology may never be able to cope with large lesions. Lesions created by FUS are usually small at about 2 mm in diameter and 200 mm^3^ in volume. For certain lesions and epileptogenic networks to be adequately treated, larger ablation volumes may be required. This may require technological advances and modifications before standard clinical utility can be achieved [114].

One of the significant limitations of MRgFUS is that the thalamotomy procedure must be unilateral, due to concerns about dysarthria resulting from bilateral thalamotomy. Further, when the lesional temperature cannot be achieved, MRgFUS is prone to failure. In comparison to VIMs or hypothalamic hamartomas, structures such as the GPi and hippocampus may be more difficult to treat because they may have fewer active ultrasound transducer elements [115].

However, there are several advantages including non-invasiveness, continuous intraoperative, monitoring of symptoms and images, fine adjustment of the target, distinct lesion borders, intraoperative physiological confirmation, real-time monitoring of the lesion process with MRI and thermometry, and immediate results.

Lipsman and colleagues treated patients suffering from symptoms of chronic ET resistant to medical therapy with tcMRgHIFU [116]. These patients were treated with thalamic ablation, which resulted in a reduction of 81.3% in tremor scores after three months.

In 2014, Magara et al. reported on the results of pallidothalamic tractotomy for thirteen patients with Parkinsons disease using MRgHIFU [117]. An assessment was conducted using the Unified Parkinson's Disease Rating Scale (UPDRS) and global symptom relief (GSR). On T2-weighted images, visible ablated lesions were observed with thermal ablation repeated up to five times. The UPDRS and GSR were reduced in these patients by 60.9% and 56.7%, respectively.

According to Jung et al. MRgFUS is effective in treating medically refractory OCD [118]. Four patients underwent bilateral anterior limb capsulotomies with favorable results. MRgFUS was evaluated in a clinical trial on ten patients for its feasibility, safety, and initial efficacy.

### 3.11. Blood-Brain Barrier Opening

The delivery of chemotherapeutic agents, antibodies, growth factors, or genes to the targeted area of the brain has been demonstrated in several animal studies [119].

As a result of FUS therapy for Alzheimer's disease (AD), several preclinical studies have demonstrated the effectiveness of delivering anti-amyloid antibodies and other disease-modifying drugs across the BBB [120]. In a phase II clinical trial of Alzheimer's patients, Mehta et al. reported that focused ultrasound-induced blood-brain barrier opening was performed safely in three patients without any adverse effects. (ClinicalTrials.gov identifier, NCT03671889) [121].

## 4. Other Lesioning

### 4.1. Radiofrequency

#### 4.1.1 Advantages

Advantages include a distinct border around the lesion during surgery and the physiological changes can be assessed, there is no restriction on the treatment area, and you can achieve results quickly [122].

#### 4.1.2 Limitations

Limitations include the risk of intracerebral hemorrhage and accuracy loss is present during surgery. Further, there is no guarantee as to the size and shape of the lesions. Consequently, large volumes must be ablated with multiple passes [123].

### 4.2. Radiosurgery

#### 4.2.1 Advantages

Advantages include potential avoidance of surgery, decreased restriction on the treatment area, ablation of a large volume of tissue, conforming to complex geometries of the lesion, and increased plasticity due to a slower radiobiologic effect [124].

#### 4.2.2 Limitations

Limitations include the length of time before visible effects, less demarcation of lesion borders and doses falling off more gradually, increased exposure to ionizing radiation, and no intraoperative feedback [125].

### 4.3. Brain-Computer Interface

Brain-computer interface (BCI) is an emerging and exciting topic in the field of neuroscience. It is defined as a system that receives neurological input from the central nervous system (CNS) and translates it into intelligible commands that an output device can act upon [126]. There are two main methods of BCI devices that are used for signal acquisition: invasive and non-invasive [127],[128]. Invasive types include implanted intracortical microelectrode arrays or electrocorticography (ECoG) where the skull must be opened to access the area of interest. Non-invasive types include electroencephalogram (EEG), magnetoencephalography (MEG), functional magnetic resonance imaging (fMRI), and positron emission tomography (PET) [128]. EEGs are beneficial in that they are relatively cheap, non-invasive, and safe to use. However, their limitations include signal attenuation due to the multitude of layers the signal must pass through to reach the EEG [129]. Compared to EEG, ECoG has superior resolution due to its proximity to the source as well as less extraneous noise from motion artifacts such as blinking [128],[130]. BCI is generally composed of 4 stages: signal acquisition, feature extraction, feature translation, and device output. Signal acquisition is the measurement of neurological input from the brain through sensory devices (EEG or microelectrode arrays). Feature extraction is the process of inspecting incoming signals and differentiating relevant information from unwanted superfluous background noise. Feature translation is defined as feeding the resulting signal into an algorithm to convert it to commands comprehensible to the output device. The device output is the final step that operates the external device to provide the response [127],[130].

Current FDA-approved DBS systems utilize an open loop system where stimulation is delivered at a constant rate during the time the device is operating. If necessary, adjustments to the signal such as a change in amplitude or frequency require manual physician intervention and can be a burden to the patient. BCI has made significant advancements in its ability to have a closed loop, where sensors collect data from the patient and automatically make adjustments without the need for clinical intervention [131]. One study was able to use a bidirectional deep brain-computer interface (dBCI) to analyze and decrypt neural inputs from brain activity and subsequently optimize control policies for motor behaviors that are tailored to a specific PD patient. They demonstrated that STN oscillopathy is a dependable neural input for closed-loop DBS using dBCI for the treatment of bradykinesia in PD [132]. Closed-loop BCI with predictive neuromodulation has also been used to treat MDD with improved efficacy compared to standard non-BCI neuromodulation methods. The system predicts the non-linear and multiband neurodynamics in MDD and can manage and control the diseased neural dynamics to effectively produce a therapeutic output signal [133]. Additionally, BCI has been shown to improve ET outcomes, as the use of dBCI in ET has diminished the total stimulation applied since it can disable neural stimulation when the patient is not actively using their limbs. This leads to an improvement in the battery life as the battery is not continuously in use at all times.

## 5. Conclusion

DBS, LS, and FUS have been demonstrated to be effective tools for the treatment of neurological disorders. The various mechanistic targets, such as the STN, GPi, VIM, and ALIC were discussed in the context of utilization and recent discoveries. In this review, we have displayed the overlap of these mechanistic targets of each procedure in many conditions such as Parkinsons Disease (PD), Essential Tremor (ET), Obsessive Compulsive Disorder (OCD), Tourette's Syndrome (TS), and Major Depressive Disorder (MDD). We explained the novel utilization of these procedures and their significant effectiveness as therapeutics for these disorders. Further, a comparison of these procedures' advantages, disadvantages, and limitations shows the need for further research to find optimal targeting. Additionally, BCI was discussed in the context of new advancements and its potential in improving QOL providing another route to treating CNS disorders.
